# Effects of a Home-Based Exercise Intervention (E-Fit) on Bone Density, Muscle Function, and Quality of Life in Girls with Adolescent Idiopathic Scoliosis (AIS): A Pilot Randomized Controlled Trial

**DOI:** 10.3390/ijerph182010899

**Published:** 2021-10-17

**Authors:** Rufina Wing-Lum Lau, Ka-Yee Cheuk, Bobby Kin-Wah Ng, Elisa Man-Shan Tam, Alec Lik-Hang Hung, Jack Chun-Yiu Cheng, Stanley Sai-Chuen Hui, Tsz-Ping Lam

**Affiliations:** 1 School of Medical and Health Sciences, Tung Wah College, Hong Kong, China; rufinalau@twc.edu.hk; 2 SH Ho Scoliosis Research Lab, Joint Scoliosis Research Center of The Chinese University of Hong Kong and Nanjing University, Department of Orthopaedics and Traumatology, Faculty of Medicine, The Chinese University of Hong Kong, Hong Kong, China; kayee607@yahoo.com (K.-Y.C.); bobng@ort.cuhk.edu.hk (B.K.-W.N.); tam.elisa@gmail.com (E.M.-S.T.); lhhung@ort.cuhk.edu.hk (A.L.-H.H.); jackcheng@cuhk.edu.hk (J.C.-Y.C.); 3 Department of Sports Science and Physical Education, Faculty of Education, The Chinese University of Hong Kong, Hong Kong, China; hui2162@cuhk.edu.hk

**Keywords:** home-based exercise intervention, high-intensity interval training, bone density, physical activity, self-image, adolescent idiopathic scoliosis

## Abstract

Background: Adolescent idiopathic scoliosis (AIS) patients have lower physical activity levels than normal adolescents, and there is an association with poorer bone and muscle health. This study evaluated the effects of a home-based exercise intervention (E-Fit) on bone mineral density (BMD), muscle function, and quality of life (QoL) in AIS-affected girls. Methods: A total of 40 AIS females aged 11 to 14 years were randomly assigned to the E-Fit or control group. The E-Fit group performed modified 7-min high-intensity interval training (HIIT) 5 days per week for 6 months. Outcome measures including BMD using dual-energy X-ray absorptiometry (DXA), muscle strength and endurance tests, physical activity levels, and QoL using self-reported questionnaires were assessed at baseline and at 6-month and 12-month follow-up. Results: In total, 14 patients in the E-Fit and 16 in the control group completed the study. The E-Fit group showed a marginally significant interaction effect in the whole body areal BMD at the 6- (*p* = 0.096) and 12-month follow-ups (*p* = 0.085). The left arm lean mass in the E-Fit group showed a statistically significant interaction effect between the 6- and 12-month follow-ups (*p* = 0.046). The E-Fit group showed improvements in physical activity participation, as measured by the Modified Baecke Questionnaire (MBQ), with a significant interaction effect in work index (*p* = 0.043), sport index (*p* = 0.050), and total score (*p* = 0.016) from baseline to the 12-month follow-up. Improvement on self-image were noted in E-Fit group across time. Conclusions: The present results provided some evidence to support the positive benefits of E-Fit for bone health and muscle function in AIS girls.

## 1. Introduction

Adolescent idiopathic scoliosis (AIS) is a complex three-dimensional spinal deformity that most commonly occurs in females aged between 10 and 16 years during the peri-pubertal growth spurt [1]. AIS is reported to be associated with low body weight, tall stature, longer arm span, lower body mass index (BMI), and delayed onset of menarche [2]. A large-scale screening program in Hong Kong has been underway since 1995. Over 800,000 students have been assessed, demonstrating a prevalence of 4.5%, which is higher than previously reported incidence of 2–4% among local Chinese girls during the peri-pubertal period [3]. When AIS is untreated or improperly treated, the curve may deteriorate, leading to functional disabilities and morbidities in adolescents.

Various studies have documented that approximately 60% of female adolescents with AIS have low bone mineral density (BMD) and more than 30% have a Z-score of less than −1 when compared with the age-/sex-matched and adjusted normal controls [4,5]. Our research group was the first to report osteopenia as one of the objective prognostic factors in predicting scoliotic curve progression [5]. In our previous longitudinal follow-up study on 327 girls with AIS, they received no intervention for their AIS and were followed up at for least 6 years until skeletal maturity. About 85% were found to have persistent osteopenia [6]. Female adolescents with AIS have also been found to have lower body weight and body mass index (BMI), with decreased body fat and fat-free mass [7,8]. The association between altered body composition and AIS has been demonstrated by Clark et al. [9] using the Avon Longitudinal Study of Parents and Children (ALSPAC). This study showed that after adjusting for the confounders, per standard deviation decrease in the lean mass at age 10 years was associated with a 20% higher risk of scoliosis, and a per standard deviation decrease in the fat mass with a 13% higher risk [8]. Girls with AIS have been known to have lower physical activity levels than their healthy peers. Low physical activity levels were significantly correlated with both areal and volumetric bone mineral density (BMD) in the spine and both hips [1]. Lower physical activity levels in girls with AIS were associated with lower cortical area and cortical bone volumetric BMD at the non-dominant distal radius [10]. Moreover, juvenile females with AIS were found to be unhappy with their lives, with more physical complaints, lower self-esteem, and a higher depression score when compared to the age-matched general norms [11].

Regular exercise during adolescence results in metabolic, physiological, neuromuscular, and psychosocial benefits, and these gains seem to extend into adulthood. Regular exercise is believed to increase bone mineral accrual and muscle mass, and enhance peak bone mass (PBM). It may thus improve muscle function and performance, while reducing the risk of osteoporosis and osteoporosis-related fractures in later life [12,13]. Exercise also exerts positive psychosocial benefits through reducing anxiety, depression, and negative mood, and improving self-esteem and cognitive function [14]. A meta-analysis of six selected studies with high methodological quality on the PEDro scale showed that physiotherapeutic scoliosis-specific exercises could prevent curve progression and improve the quality of life in patients with AIS [15]. A recent controlled study identified that younger patients with a lower Risser grade were most likely to respond positively to exercise therapy in terms of curve improvement and stability, and an early conservative exercise intervention for the patients with AIS was beneficial [16]. Nevertheless, these scoliosis-specific exercises require close supervision from on-site experts, repeated clinical visits, and lengthy sessions.

The E-Fit exercise intervention (E-Fit) is specifically designed to address these shortfalls and is aimed at bone health, muscle function, and quality of life for AIS patients as a feasible adjunct conservative treatment. The E-Fit is a modification of 7-min high-intensity interval training (HIIT), which is a well-received exercise method with proven significant training effects on fitness. It is particularly suitable and safe for young people [17]. It comprises a board range of high-impact weight-bearing exercises with frequent intermittent rest intervals in between each exercise. These exercises are performed in varying speeds and directions so that significant stimulation is provided to improve bone quality and muscle mass [13]. To encourage our subjects to exercise safely, all exercises in E-Fit can be performed in a small home environment with minimal equipment, and some exercises such as jumping jack and step-ups onto a stool were modified to avoid excessive stress onto the spinal column and peripheral joints. To provide fun and minimize boredom, a pool of 40 video clips of individual exercises was designed, and a web-based computer program was developed to generate a randomized 7-min E-Fit of 12 different exercises each time. Each exercise lasted for 30 s with a 10 s rest interval in between each exercise. E-Fit is also time-efficient and focuses on cultivating exercise habits and remaining active in the home environment during the early stage of diagnosis.

The aim of the current pilot study was to determine the feasibility of a 6-month home-based E-Fit exercise intervention for skeletally immature female adolescents with AIS. We also investigated the effect of this program on their bone density, muscle function, and quality of life.

## 2. Materials and Methods

### 2.1. Subjects

A total of 40 female adolescents aged between 11 to 14 years with AIS were targeted and recruited from an out-patient scoliosis clinic at a local hospital. All subjects were included if they were female AIS patients who were newly diagnosed with AIS by standard standing long X-ray examinations with a Cobb angle larger or equal to 15°, had not received prior treatment for their AIS, and been cleared for physical activity by their doctors. Subjects were excluded if they (i) had a Cobb angle larger or equal to 40°, (ii) had scoliosis with any known etiologies such as congenital, neuromuscular, metabolic, and skeletal dysplasia, (iii) had known endocrine and connective tissue abnormalities, (iv) had a known heart condition or other diseases that could affect the safety of exercise, (v) had eating disorders or gastrointestinal malabsorption disorders, and (vi) were currently taking medications affecting their bone or muscle metabolism. All subjects signed a consent form in the presence of their parents after thorough explanation by the attending doctor. No similar study of this nature has been reported in the literature. We assumed a large effect size of 0.85, statistical power of 0.8, and a level of significance at 0.05. A sample size of 19 subjects was thus required in each group.

The 40 subjects completed the baseline measurements and were randomly assigned to the E-Fit (*n* = 20) or the control group (*n* = 20) by drawing lots. The E-Fit group performed modified 7-min high-intensity interval training (HIIT) for 6 months administered through an integrated application of an exercise provision website and a mobile app. The compliance of exercise intervention was monitored by using an accelerometer and a self-reported check diary booklet. The physical activity movement was recorded at real-time when performing E-Fit, and the movement signals represented as activity counts were transmitted to the mobile app automatically for records. The participant would carry out the E-Fit 5 days per week, with the remaining 2 days as rest days. To increase exercise compliance, parents were encouraged to actively support the subjects in performing E-Fit. Research personnel also contacted subjects and their parents by phone calls on regular basis to encourage compliance with the exercise intervention and to resolve any problems encountered. The control group had no intervention and received only standard care.

### 2.2. Measurements

At baseline, anthropometric and sexual maturity measurements as well as clinical features were obtained. Body height, weight, sitting height, and arm span were measured with standard stadiometry techniques. Sexual maturity level including onset of menarche, breast development, and pubic hair distribution was graded with a standard Tanner Scale using established and validated protocols [18]. Clinical features of scoliosis in terms of Cobb angle were assessed by a long standard standing posteroanterior (PA) whole-spine X-ray using the standard Cobb method for grading curve severity.

The following outcome measurements were conducted at baseline, 6 months after completion of E-Fit, and at 12-month follow-up. Areal BMD (g/cm^2^) and bone mineral content (BMC) (g/cm) of the non-dominant femoral neck, whole body BMD, and muscle mass by whole body less head (WBLH) were measured by dual energy X-ray absorptiometry (DXA) (Horizon, Hologic Inc., Bedford, MA, USA). Standardized scanning procedures provided by the manufacturer were followed to ensure unified and comparable measurements. Radiation doses associated with DXA are very low, with the effective doses <3uSv [19]. Quality assurance was performed by daily calibration against the standard phantoms provided by the manufacturer, with a precision error of 1.9% for BMD value for patient scan and <1.0% for whole body phantom scan. The spinal BMD was not measured because this value is confounded by the rotation the spine in AIS patients, which is one of typical characteristics in scoliosis curvature [20]. It has been shown that BMD of the hips correlates with the spinal BMD in normal subjects [4].

Muscle endurance of the legs, back, and trunk was assessed by isometric squat endurance test, the Biering–Sorensen test, and isometric curl-up test with standardized procedures. The timing started when subjects were in the standard positions and was stopped when they could not maintain the position. The time in seconds in which the participant maintained the position was recorded.

The quality of life (QoL) of the subjects was assessed with the Chinese version of The Scoliosis Research Society-22r Questionnaire (SRS-22r). The SRS-22r is an internationally validated questionnaire that contains 22 questions organized in 5 domains covering different aspects of the QoL of patients with scoliosis [21].

Compliance with the E-Fit during the 6-month period was monitored by an accelerometer worn on the wrist and a self-reported diary. The accelerometers were collected at the 6-month follow-up visit after completion of E-Fit. The physical activity level was also assessed using Modified Baecke Questionnaire (MBQ) adapted from Pols et al. [22].

Dietary intakes were evaluated using a modified Food Frequency Questionnaire (FFQ) based on the data obtained and validated in the Hong Kong Adult Dietary Survey [23]. Subjects were asked about their usual frequency of consumption in the past 12 months of foods from the food list. Daily nutrient intake was calculated by the Food Processor Nutrition analysis and Fitness software version 8.0 (Esha Research, Salem, MA, USA), with the addition of composition of some local foods based on a food composition table from China. To collate the feedback of the study, a feedback questionnaire was presented to subjects of both groups and their parents at the 6-month follow-up visit.

### 2.3. Statistical Analyses

Data were checked with Levene’s test for normality and equality of variances prior to analyses. Baseline characteristics of the subjects of both E-Fit and control groups including age, anthropometric and sexual maturity measurements, and clinical features of scoliosis were compared using the independent Student’s t-test and reported as means and standard deviations. Two-way repeated-measures analysis of covariance (ANCOVA) using age and body mass index as covariates were conducted to compare the differences in the BMD, muscle mass, muscle function, and curve severity measurements across time (i.e., at baseline, 6 months after completion of the E-Fit, and at 12-month follow-up) between the E-Fit and control groups. Post-hoc pair-wise analyses were adjusted with Bonferroni correction based on a single Group (2 levels) x Time (3 levels) model to obtain individual p values. Covariates of age, body mass index, breast development, and pubic hair development graded by the Tanner Scale and baseline measurements were included in the model where appropriate. The statistical significance level was set at *p* = 0.05.

## 3. Results

### 3.1. Group Comparison

**Figure 1:**
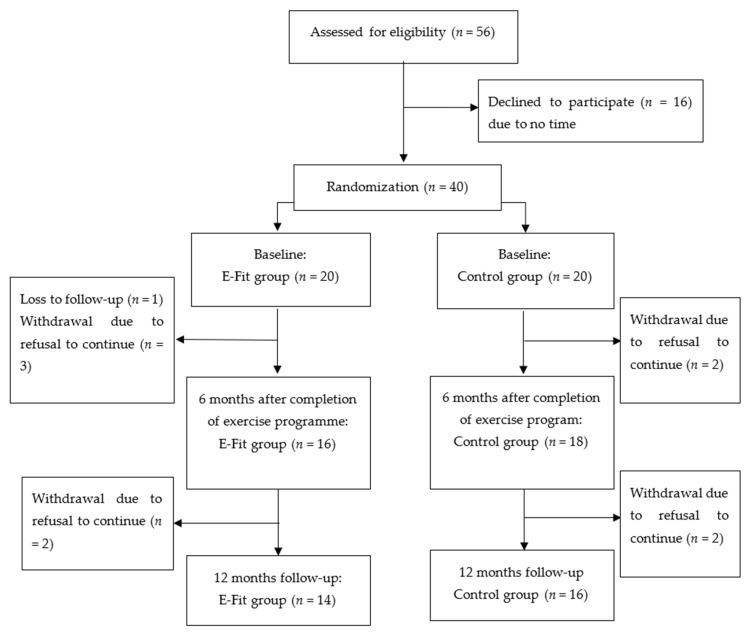
Flow chart of the study design.

A total of 56 adolescents were approached, and 40 of them agreed to participate in the study. These 40 adolescents were randomly assigned into either the E-Fit (*n* = 20) or control (*n* = 20) groups. A total of 34 subjects (*n* = 16 in the E-Fit group and *n* = 18 in control group) completed the study and returned for the 6-month post-exercise follow-up. The post-exercise dropout rate was 15%. At 12-month follow-up, only 30 subjects returned (*n* = 14 in E-Fit and *n* = 16 in control group) (Figure 1). The loss to follow-up rate was 25%. No significant group difference was found regarding the Cobb angle and vitamin D and calcium levels from baseline to the 6- and 12-month follow-up. At baseline, 7 subjects (35%) in the E-Fit group and 7 subjects (35%) in the control group required bracing treatment. A total of 5 subjects (36%) in the E-Fit and 8 subjects (50%) in the control group were on bracing treatment at 12-month follow-up. The subjects in both groups had similar baseline characteristics, as shown in Table 1 (all *p* > 0.05).

### 3.2. Treatment Outcomes on Bone and Muscle Parameters

The results of the bone mineral density, bone mineral content, and muscle endurance measures in both groups are shown in Table 2. At the completion of the 6-month intervention, the E-Fit group showed an increase in the whole-body areal BMD with a marginal significant interaction effect (*p* = 0.096) at 6-month follow-up, and the improvement was maintained from baseline to 12-month follow-up (*p* = 0.085). The increase in left femoral neck BMC in E-Fit was smaller when compared to the control group, with a significant interaction effect (*p* = 0.021). The left arm lean mass in the E-Fit group showed a statistically significant interaction effect between the 6- and 12-month follow-up (*p* = 0.046). For the muscle strength and endurance measurements, the E-Fit group showed a positive trend in the isometric curl-up test when compared to the control group between the 6- to 12-month follow-ups (*p* = 0.095), but the difference was not sustained at the 12-month follow-up.

### 3.3. Treatment Outcomes on Physical Activity Level and Quality of Life Measures

The physical activity levels continuously improved in the E-Fit group, with a significant interaction effect across the three time-points in terms of their work index (*p* = 0.043), sport index (*p* = 0.050), and total score (*p* = 0.016) of MBQ (Table 3). The E-Fit group also showed a marginally significant interaction effect in the self-image domain (*p* = 0.066) and so in the total score (*p* = 0.086) of the SRS-22r between the 6- and 12-month follow-ups. The interaction effect was not significant between groups from baseline to 12-months. On the contrary, the control group appeared to continuously decline, although not statistically significant, in their physical activity levels and quality of life measures across the three time points (Table 3).

### 3.4. Compliance of Exercise Intervention

Only 9 subjects (*n* = 4 in the E-Fit and *n* = 5 in the control groups) wore the accelerometer for more than 70 days throughout the study period. In total, 24 out of 40 (60%) subjects did not regularly synchronize the accelerometer with the mobile device and 13 subjects reported a loss of their accelerometer. The mean compliance recorded by the accelerometer was 15%, ranging from 0% to 64%.

### 3.5. Feedback Questionnaires

In total, 33 subjects (*n* = 15 in E-Fit group and *n* = 18 in control group) returned the feedback questionnaire. Most of them thought the accelerometer was good (67%), light (86%), and convenient to use (77%). However, 7 subjects (21%) commented that the outlook of the accelerometer was not attractive, and it was not easy to charge it or synchronize data to mobile devices. In total, 10 subjects in the E-Fit group thought the exercise videos were good because the instructions were clear (80%), exercises could be done at home easily (70%), and the exercises were interesting (40%). Subjects and parents believed that exercise compliance could be enhanced by shortening the length of intervention of exercise (47% of subjects and 38% of parents), using exercise without any equipment (33% of subjects and 53% of parents), competing with other subjects (47% of subjects), sharing with other subjects (31% of parents), and by giving a gift for the subjects who had a good compliance (31% of subjects). Six subjects who withdrew from the study suggested conducting the assessment after school or during the weekend as the assessment session was lengthy.

## 4. Discussion

To our knowledge, this study is the first clinical trial to investigate the effects of a specifically designed exercise intervention of short-duration but high-impact weight bearing exercises in female adolescents with AIS. The 6-month home-based E-Fit exercise intervention is characterized by a series of short-duration, high-impact weight-bearing exercises with frequent rest periods in between them, which is particularly suitable for the busy lifestyles of adolescents living in Hong Kong in small home environments. In the present pilot study, the E-Fit was well-received by the subjects as they claimed that they could easily follow the exercise instructions and perform the exercises at home with little hassle. No adverse events were reported by the subjects who completed the intervention. This provides support for further studies using E-Fit in future studies.

In term of bone mineral density, the E-Fit group showed a positive trend in the whole-body areal BMD after the completion of 6-month E-Fit program (Table 2). The improvement in whole-body areal BMD in the E-Fit group continued to show a marginally significant interaction effect at the 12-month follow-up when compared to the baseline (Table 2). A similar continuous improvement was also observed in the left arm lean mass in the E-Fit group at the 12-month follow-up when compared to the 6-month timepoint (Table 2). Short bouts of high-impact weight-bearing exercises seemed to induce positive physiological adaptations in skeletal muscle and bone mass in adolescents with AIS, as in other diseased populations and healthy adults/adolescents [24,25,26,27]. Patients with AIS and low bone density have at least twice the risk of curve progression [28]. The reported improvement in the BMD in the present study is of particular importance to skeletally immature adolescents with AIS in preventing or slowing down curve progression as well as the development of osteoporosis and its associated complications in adulthood [29]. Moderate-to-vigorous physical activity has recently been shown to have positive impact on bone strength accrual in adolescents [30]. However, the left femoral neck BMC in the E-Fit group showed a gentler increase from baseline to the 6-month follow-up, but the increase at the 12-month follow-up was comparable with the control. The smaller increase in the E-Fit group from baseline to 6 months might be due to the dissociation between skeletal expansion and skeletal mineralization during the active growing years in adolescence. The growth in bone size precedes increases in bone accrual [31]. Future studies could include evaluation of bone distribution, geometry, and architecture using peripheral quantitative computed tomography (pQCT) to detect smaller changes in longitudinal studies during development [32]. Given the nature of moderate-to-vigorous intensity with the E-Fit approach, it is worthwhile conducting further studies on the E-Fit to investigate its effect on bone strength accrual.

Both the E-Fit and control groups showed improvements, although they did not reach statistical significance in muscle strength across the three timepoints (Table 3). However, the E-Fit group demonstrated better improvement in the isometric curl-up test when compared to the control group between the 6- and 12-month follow-up (Table 3). Our preliminary findings support that exercise in early life can increase muscle function and performance in young people [1,26]. Our current findings also showed some potential benefits of the E-Fit in muscle endurance. Younger AIS patients receiving early exercise interventions show better control or improvement in curve progression as compared to older AIS patients [16]. As more rapid changes in curve progression are usually found during periods of growth spurts and especially at the beginning of puberty, the E-Fit could be used as an adjunct conservative intervention to conventional clinic-based regimens at the early stage of diagnosis for AIS patients at a younger age to better control or improve musculoskeletal health. Since dietary intake can influence body metabolism, this pilot study has considered the dietary profiles of both group to eliminate confounding metabolic variables, and no significant group difference was found.

**Figure 2:**
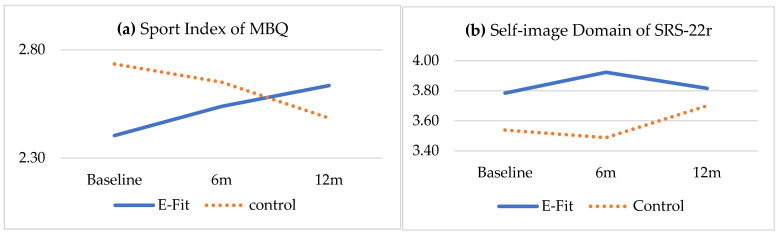
Changes of (**a**) sport index of MBQ and (**b**) self-image domain of SRS-22r across groups and time in the subjects.

For quality of life, the E-Fit group showed a trend of improvement in the self-image domain and hence in the total score of the SRS-22r from the 6- to 12-month follow-ups (Table 3 and Figure 2). The insignificant results in terms of quality of life might be due to a ceiling effect as the SRS-22r score was already quite high in both groups at baseline (Table 3). It is worth noting that the improvement in the self-image domain of the SRS-22r and the positive feedback from the subjects about the E-Fit may reflect some degree of perceived enjoyment of the E-Fit exercises. Moreover, the E-Fit group showed a continuous improvement, with a significant interaction effect in their physical activity level in the domains of work index, sport index, and the total score in the MBQ from the baseline to 6- and 12-month follow-ups (Table 3 and Figure 2). In contrast, the control group showed a decline in physical activity level over all time points. These findings are in line with previous studies which showed that lower physical activity levels and lower quality of life are commonly reported among patients with AIS as compared with their healthy peers [11]. It was found that they tend to overestimate the severity of their scoliosis. The perception of their body schema and spinal curvature can be improved by enhancing motor skills through participation in physical activity [33]. The potential psychological benefits brought about by the E-Fit came as a surprise in the present study. The E-Fit appears able to encourage more habitual physical activity in our relatively inactive sample group. Performing more active exercises in turn may generate a better self-image, relieve stress, and promote a healthy lifestyle in the study subjects [15,21].

There are several limitations of this pilot study. Firstly, compliance regarding accelerometer use was very low, which posed challenges to monitoring exercise compliance and intensity in the subjects. Using an accelerometer with automatic data synchronization and modifying the wear protocol may result in a more accurate record of compliance data. The current study used only DXA to evaluate bone density, but future studies could include the evaluation of bone geometry and architecture measurements with pQCT to assess the effect of physical activity on bone health. The sample size was small in this study, which might explain the lack of treatment effects in certain outcome measures. However, the present preliminary data provide us with more information about the sample sizes for future studies on the E-Fit. We were unable to quantify the actual exercise intensity of our subjects. Laboratory exercise testing can be used in future studies to determine the physiological responses and optimal intensity with regard to the E-Fit exercise intervention.

## 5. Conclusions

Our preliminary results indicated that our specifically designed E-Fit exercise intervention for female adolescents with AIS showed benefits, especially regarding bone and muscle function, physical activity levels, and the quality of life of the study subjects. The moderate-to-high impact weight-bearing exercises of the E-Fit appear to be safe and feasible for the home environment. The E-Fit might be an adjunct to conventional clinical-based exercise interventions for early scoliosis. Further studies are needed to determine the physiological responses and optimal dosage of the E-Fit exercise intervention.

## Figures and Tables

**Table 1:** Basic characteristics of the E-Fit and control group at baseline.

Basic Characteristics	E-Fit (*n* = 20)	Control (*n* = 20)	*p*-Value
Age (year)	12.8 ± 0.9	13.2 ± 1.1	0.178
Menarchal age (year)	11.5 ± 1.1	11.7 ± 0.9	0.441
Tanner Scale: Breast development (1–5)	2.9 ± 0.7	2.8 ± 0.8	0.687
Tanner Scale: Pubic hair distribution (1–5)	2.2 ± 0.8	2.4 ± 0.7	0.389
Height (cm)	156.2 ± 8.1	154.9 ± 8.5	0.620
Arm span (cm)	155.3 ± 8.8	153.1 ± 9.1	0.432
Weight (kg)	45.8 ± 10.7	42.7 ± 6.6	0.278
Body mass index (kg/m^2^)	17.5 ± 5.2	18.2 ± 2.5	0.586
Cobb angle (°)	20.6 ± 5.0	23.4 ± 6.7	0.178
Bracing	7 (35%)	7 (35%)	-

The values are presented as mean ± standard deviation or % as appropriate.

**Table 2:** The means of bone mineral density, bone mineral content, and muscle endurance measures over time of the subjects.

	E-Fit	Control	Time * Group Interaction (*p*-Value)			
	**(*n* = 14)**	**(*n* = 16)**	**A vs. B**	**B vs. C**	**A vs. C**	**A vs. B vs. C**
**Whole-body aBMD (g/cm^2^) ^a^**						
A: Baseline	0.880 ± 0.105	0.909 ± 0.127	0.096 ^#^			
B: 6 months post-exercise	0.899 ± 0.106	0.907 ± 0.105		0.572		
C: 12 months	0.947 ± 0.099	0.958 ± 0.101			0.135	0.085 ^#^
**Left femoral neck BMC (g/cm) ^b^**						
A: Baseline	2.952 ± 0.497	3.040 ± 0.645	0.021 *			
B: 6 months post-exercise	3.022 ± 0.447	3.212 ± 0.635		0.221		
C: 12 months	3.267 ± 0.504	3.365 ± 0.615			0.726	0.216
**Right femoral neck BMC (g/cm) ^b^**						
A: Baseline	3.072 ± 0.481	3.121 ± 0.644	0.627			
B: 6 months post-exercise	3.163 ± 0.445	3.218 ± 0.571		0.651		
C: 12 months	3.375 ± 0.458	3.397 ± 0.595			0.836	0.891
**Left arm lean muscle mass (kg) ^b^**						
A: Baseline	1.321 ± 0.262	1.260 ± 0.190	0.492			
B: 6 months post-exercise	1.352 ± 0.270	1.297 ± 0.176		0.046 *		
C: 12 months	1.414 ± 0.231	1.307 ± 0.163			0.084 ^#^	0.083 ^#^
**Isometric curl-up test (seconds) ^c^**						
A: Baseline	100.21 ±138.78	51.38 ± 47.65	0.13			
B: 6 months post-exercise	129.50 ± 169.99	45.44 ± 24.22		0.095 ^#^		
C: 12 months	115.93 ± 158.76	89.75 ± 127.98			0.130	0.316

The values are presented as mean ± standard deviation. Covariates: ^a^ age, pubic hair and breast development; ^b^ age and BMI; ^c^ age and BMI and isometric curl-up test at baseline. Abbreviations: aBMD = areal bone mineral density; BMC = bone mineral content. * *p* ≤ 0.05; ^#^ *p* ≤ 0.10.

**Table 3:** The means of physical activity level and quality of life measures over time of the subjects.

	E-Fit	Control	Time * Group Interaction (*p*-Value)			
	**(*n* = 13)**	**(*n* = 16)**	**A vs. B**	**B vs. C**	**A vs. C**	**A vs. B vs. C**
**Physical activity level by Modified Baecke Questionnaire (MBQ)**						
***Work index***						
A: Baseline	2.18 ± 0.29	2.45 ± 0.29	0.101			
B: 6 months post-exercise	2.15 ± 0.29	2.24 ± 0.16		0.330		
C: 12 months	2.28 ± 0.29	2.27 ± 0.20			0.019 *	0.043 *
***Sport index***						
A: Baseline	2.40 ± 0.51	2.73 ± 0.64	0.410			
B: 6 months post-exercise	2.54 ± 0.56	2.63 ± 0.66		0.158		
C: 12 months	2.63 ± 0.59	2.48 ± 0.72			0.016 *	0.050 *
***Leisure index***						
A: Baseline	2.33 ± 0.38	2.39 ± 0.42	0.589			
B: 6 months post-exercise	2.51 ± 0.33	2.42 ± 0.52		0.918		
C: 12 months	2.44 ± 0.36	2.33 ± 0.44			0.367	0.488
***Total score***						
A: Baseline	6.92 ± 0.80	7.57 ± 0.86	0.128			
B: 6 months post-exercise	7.20 ± 0.99	7.28 ± 1.05		0.210		
C: 12 months	7.35 ± 0.86	7.08 + 1.04			0.009 *	0.016 *
**Quality of life by SRS22r questionnaire [5 Best–1 Worst]**						
***Self-image***						
A: Baseline	3.78 ± 0.59	3.54 ± 0.44	0.480			
B: 6 months post-exercise	3.92 ± 0.44	3.49 ± 0.62		0.066 ^#^		
C: 12 months	3.82 ± 0.56	3.70 ± 0.61			0.534	0.272
***Total***						
A: Baseline	4.40 ± 0.25	4.27 ± 0.40	0.650			
B: 6 months post-exercise	4.38 ± 0.26	4.16 ± 0.40		0.086 ^#^		
C: 12 months	4.31 ± 0.23	4.21 ± 0.45			0.835	0.639

The values are presented as mean ± standard deviation. Covariates: age and BMI. * *p* ≤ 0.05; ^#^ *p* ≤ 0.10. Remarks: There is one missing data each for MBQ and SRS-22r in E-Fit group.
